# Allopregnanolone Effects on Inhibition in Hippocampal Parvalbumin Interneurons

**DOI:** 10.1523/ENEURO.0392-22.2023

**Published:** 2023-03-10

**Authors:** Xinguo Lu, Peter Lambert, Ann Benz, Charles F. Zorumski, Steven J. Mennerick

**Affiliations:** 1 Department of Psychiatry, Washington University in St. Louis, St. Louis, MO 63110; 2 Department of Neuroscience, Washington University in St. Louis, St. Louis, MO 63110; 3 Taylor Family Institute for Innovative Psychiatric Research, Washington University in St. Louis, St. Louis, MO 63110

**Keywords:** antidepressant, inhibition, interneuron, neurosteroid

## Abstract

Allopregnanolone (AlloP) is a neurosteroid that potentiates ionotropic GABAergic (GABA_A_) inhibition and is approved for treating postpartum depression in women. Although the antidepressant mechanism of AlloP is largely unknown, it could involve selective action at GABA_A_ receptors containing the δ subunit. Despite previous evidence for selective effects of AlloP on α4/δ-containing receptors of hippocampal dentate granule cells (DGCs), other recent results failed to demonstrate selectivity at these receptors (Lu et al., 2020). In contrast to DGCs, hippocampal fast-spiking parvalbumin (PV) interneurons express an unusual variant partnership of δ subunits with α1 subunits. Here, we hypothesized that native α1/δ receptors in hippocampal fast-spiking interneurons may provide a preferred substrate for AlloP. Contrary to the hypothesis, electrophysiology from genetically tagged PV interneurons in hippocampal slices from male mice showed that 100 nm AlloP promoted phasic inhibition by increasing the sIPSC decay, but tonic inhibition was not detectably altered. Co-application of AlloP with 5 μm GABA did augment tonic current, which was not primarily through δ-containing receptors. Furthermore, AlloP decreased the membrane resistance and the number of action potentials of DGCs, but the impact on PV interneurons was weaker than on DGCs. Thus, our results indicate that hippocampal PV interneurons possess low sensitivity to AlloP and suggest they are unlikely contributors to mood-altering effects of neurosteroids through GABA effects.

## Significance Statement

Allopregnanolone (AlloP) is an endogenous positive allosteric modulator of GABA_A_ receptors and appears to have antidepressant effects triggered faster than traditional medications. The antidepressant mechanism of AlloP is largely unknown and we tested whether inhibition of parvalbumin (PV) interneurons in mouse hippocampus could participate. Our results indicate that PV interneurons possess low sensitivity to AlloP despite favorable receptor subunit composition.

## Introduction

Inhibition sculpts neurophysiological excitability and modulates oscillations in brain networks that give rise to sensation, perception, and cognition. In the mammalian CNS, most inhibition is mediated by GABA_A_ receptors, which are ligand-gated, chloride-permeable ion channels (Sigel and Steinmann, 2012). They are heteropentameric and composed of two α, two β, and a fifth subunit, usually either δ (extrasynaptic in certain cells) or γ2 (synaptic in most cells; Pirker et al., 2000; Wei et al., 2003). Subunits are assembled according to precise rules. For instance, δ subunits preferentially co-assemble with α4 or α6 subunits in a select group of projection cell types, including dentate granule cells (DGCs) of the hippocampus (α4 pairing). These δ-containing receptors localize extrasynaptically, have high GABA sensitivity, and mediate tonic inhibition (Brown et al., 2002; Wei et al., 2003; Glykys et al., 2008) and some slow phasic inhibition (Herd et al., 2013; Sun et al., 2018; Shu et al., 2021). GABA is the neurotransmitter agonist of GABA_A_ receptors and is released by GABAergic interneurons. Although perhaps underappreciated, GABAergic interneurons themselves express GABA_A_ receptors and receive phasic and tonic inhibition from other interneurons (Pelkey et al., 2017). In contrast to more extensively studied projection cells, hippocampal interneurons, especially fast-spiking parvalbumin-positive (PV) interneurons, express an unusual variant partnership of δ subunits with α1 subunits (McDonald and Mascagni, 2004; Milenkovic et al., 2013; Hu et al., 2014).

Allopregnanolone (AlloP) is an endogenous neurosteroid that functions as a positive allosteric modulator to potentiate GABAergic inhibition (Harrison et al., 1987). AlloP, formulated as brexanolone/Zulresso, was recently approved for treating postpartum depression in women and has shown promise in major depressive disorder (Kanes et al., 2017; Gunduz-Bruce et al., 2019; Walton and Maguire, 2019). Although the overall benefit in humans and precise onset of any antidepressant action are still unclear, mechanisms through which AlloP act are of considerable interest, in part because onset is faster than conventional antidepressants.

A prevailing view is that neurosteroids, like 3α, 21-dihydroxy-5α-pregnan-20-one (THDOC) and AlloP, selectively potentiate δ-containing GABA_A_Rs to enhance tonic inhibition. The potentiating effect of neurosteroids is nearly abolished in δ knock-out DGCs (Stell et al., 2003; Carver and Reddy, 2016). Further, the anesthetic effects of neurosteroids are strongly altered in δ deficient mice (Spigelman et al., 2003). Finally, recent data suggest that δ-containing PV interneurons in the basolateral amygdala may be important for the mood-altering effects of neurosteroids (Antonoudiou et al., 2022). Here, we explore hippocampal PV interneurons because of the role of the hippocampus in cognitive changes associated with many neuropsychiatric disorders and the previous association with antidepressant effects.

Previous studies, using novel pharmacoresistant GABA_A_R subtypes, showed that AlloP, while indeed augmenting inhibition, does not selectively potentiate classical α4/δ-containing receptors to mediate tonic inhibition in hippocampal DGCs compared with γ2-containing receptors in the same cells (Lu et al., 2020). It is possible that noncanonical δ pairings (e.g., with α1 in PV interneurons) exhibit higher neurosteroid sensitivity than observed in DGCs. If that is the case, it could represent an important substrate of drug actions at the low concentrations thought to be therapeutically relevant.

We hypothesized that AlloP disinhibits principal cells by enhancing GABA_A_R function on PV interneurons, thereby inhibiting them and promoting excitability of principal neurons. We genetically tagged mouse PV interneurons and examined how these cells respond to AlloP during phasic and tonic inhibition. We find little evidence for AlloP potentiation of baseline tonic current and only mild potentiation of IPSCs and tonic current recorded from PV interneurons in the presence of GABA. The impact of AlloP on excitability of DGCs was stronger than on excitability in PV interneurons. By contrast, field EPSPs (fEPSPs) in CA1 were modestly potentiated by AlloP. Overall, the results preclude a large role for hippocampal interneuron GABA receptors, especially tonic inhibition in PV interneurons, in therapeutic AlloP effects.

## Materials and Methods

### Animals

**Figure 1.**
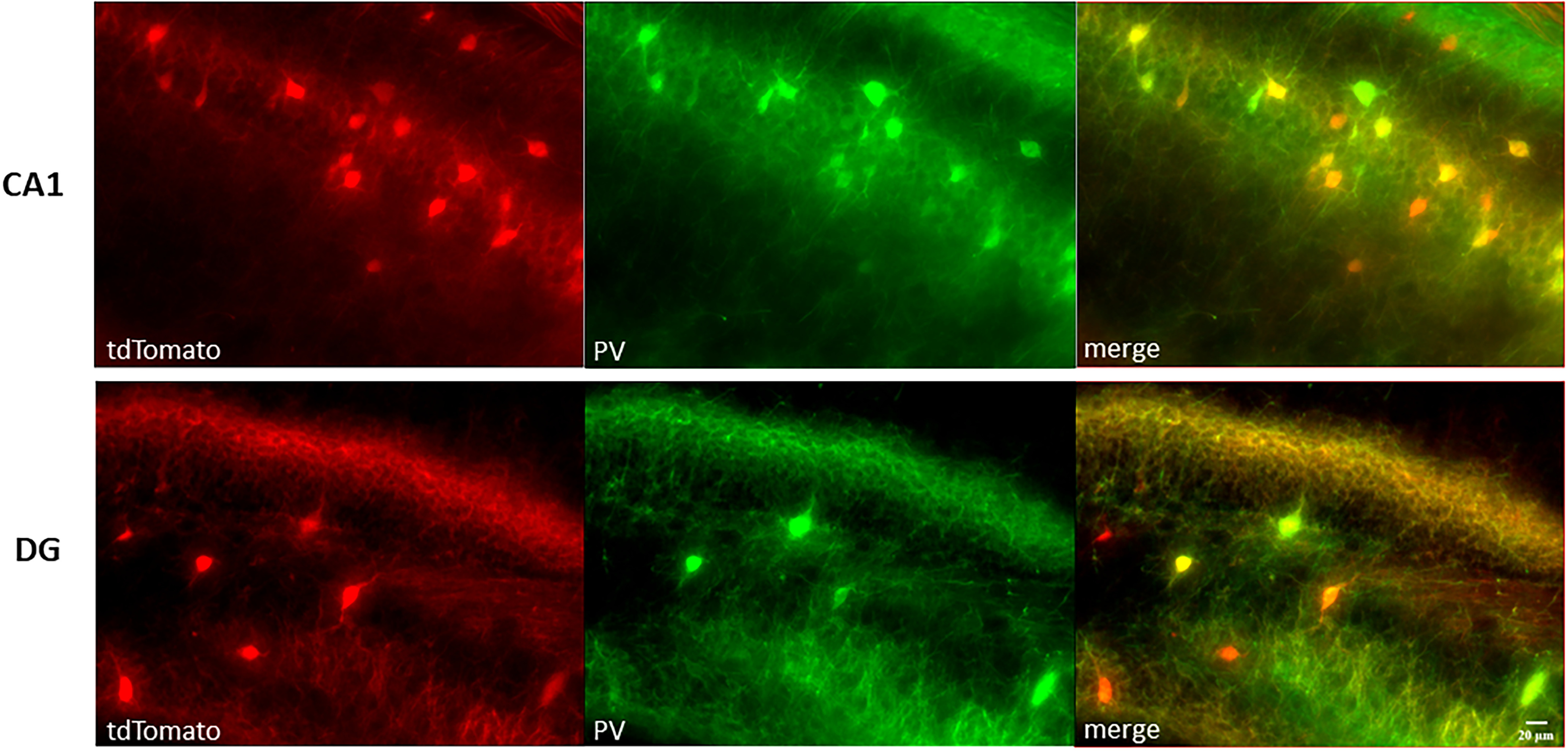
tdTomato fluorescence is a reliable indicator of PV interneurons in hippocampal CA1 and dentate gyrus from Ai14::PVCre mouse tissue. Top images, CA1. Bottom images, DG (dentate gyrus). Scale bar: 20 μm.

Male mice of PVCre or Ai14::PVCre from postnatal day (P)30 to P60 were used. We failed to find a clear change in exogenous AlloP effect with age, as demonstrated in the data available on Figshare. We also examined two female mice, whose data appeared similar to that of males and are included in the data available on Figshare. Sibling animals were group housed in zeitgeber time (ZT) 12:12 conditions, with ZT0 = 6 A.M. PV interneurons were labeled using two approaches: viral injection of pAAV5-hSyn-DIO-EGFP (Addgene, 50 457) into hippocampus of PVCre animals or Ai14::PVCre reporter mice (Ai14, Jackson Lab 007914; PVCre, Jackson Lab 017320). We assessed reporter accuracy with immunostaining against PV in Ai14 mice (Fig. 1). PV antibody (mouse anti-PV, P3088, Millipore Sigma) co-labeled 73% of tdTomato-positive cells (190 cells counted), consistent with precedent hippocampal assessments (Ledri et al., 2014). To isolate GABA current mediated by δ-containing GABA_A_ receptors, we used picrotoxin (PTX)-resistant knock-in line (δ* KI; Sun et al., 2018). To eliminate current mediated by δ-containing GABA_A_ receptors in PV interneurons, *Gabrd* floxed mice (Lee and Maguire, 2013) were bred to PVCre mice to knock out δ-containing GABA_A_ receptors [conditionally knocked out (cKO)]. In some experiments, general interneuron populations were identified by pAAV1-mDlx-NLS-mRuby2 (Addgene, 99130; Dimidschstein et al., 2016).

### Slice preparation

Mice were anesthetized with isoflurane and decapitated according to protocols approved by the Washington University IACUC. Coronal brain slices (300-μm thickness) were cut using a Leica VT1200 vibratome. During slicing, slices were maintained in ice-cold, modified NMDG-HEPES recovery artificial CSF (aCSF; in mm: 92 NMDG, 2.5 KCl, 1.25 NaH_2_PO_4_, 30 NaHCO_3_, 20 HEPES, 25 glucose, 2 thiourea, 5 Na-ascorbate, 3 Na-pyruvate, 0.5 CaCl_2_, and 10 MgSO_4_; 300 mOsm; pH 7.3–7.4). After slicing, slices recovered in modified NMDG-HEPES recovery aCSF at 32°C, and a Na^+^-rich spike-in solution (4 ml, 2 m) was added to gradually increase Na^+^ concentration to improve the success of gigaseal formation (Ting et al., 2018). After recovery, slices were stored in modified HEPES holding aCSF (in mm: 92 NaCl, 2.5 KCl, 1.25 NaH_2_PO_4_, 30 NaHCO_3_, 20 HEPES, 25 glucose, 2 thiourea, 5 Na-ascorbate, 3 Na-pyruvate, 2 CaCl_2_, and 2 MgSO_4_; 300 mOsm; pH 7.3–7.4) for at least 1 h at 25°C before experimental recording. Drugs were obtained from ThermoFisher Scientific/MilliporeSigma except where noted.

### Whole-cell recording

During recording, slices were transferred to a recording chamber with continuous perfusion (2 ml/min, 32°C) of oxygenated, regular aCSF (in mm: 125 NaCl, 25 glucose, 25 NaHCO_3_, 2.5 KCl, 1.25 NaH_2_PO_4_, equilibrated with 95% oxygen-5% CO_2_ plus 2 CaCl_2_, and 1 MgCl_2_; 310 mOsM). IR-DIC microscopy (Nikon FN1 microscope and Photometrics Prime camera) was used to visualize and identify target cells during somatic, whole-cell recording. Borosilicate glass pipettes (World Precision Instruments, Inc) with open tip resistance of 3–6 MΩ were used for whole-cell recording. Cells were recorded with a MultiClamp 700B amplifier (Molecular Devices), Digidata 1550 16-bit A/D converter, and pClamp 10.4 software (Molecular Devices). A 5-min stabilization period followed initial break-in before recordings commenced.

To record action potentials, neurons were recorded in current-clamp mode using pipettes filled with K-gluconate internal solution (in mm: 140 K-gluconate, 4 MgCl_2_, 10 HEPES, 0.4 EGTA, 4 MgATP, 0.3 NaGTP, and 10 phosphocreatine; pH was adjusted with KOH to pH 7.25; 290 mOsm). Step currents from −50 pA with increments of 20 pA were injected to measure membrane properties and to elicit action potentials. Maximum firing frequency and action potential half width were calculated using Clampfit. For percentage accommodation, the 13th to 14th action potential interval was divided by the 3rd to 4th action potential interval. For cells that had fewer than 14 action potentials, the last and first intervals were used instead. To calculate membrane resistance, the steady-state voltage change in response to −50-pA step current was measured, and resistance was calculated by Ohm’s Law.

To record phasic and tonic GABAergic current, 10 μm NBQX (Tocris Bioscience) and 50 μm D-APV (Tocris Bioscience) were added in the regular aCSF to inhibit ionotropic glutamate receptors. Pipettes were filled with cesium chloride internal solution (in mm: 130 CsCl, 10 HEPES, 5 EGTA, 2 MgATP, 0.5 NaGTP, and 4 QX-314; pH adjusted to 7.3 with CsOH; 290 mOsm). Cells were patched at −70 mV in voltage-clamp gap-free mode at 5 kHz, filtered at 2 kHz using eight-pole Bessel filter.

### Field EPSP recording

To record population spikes in CA1, a bipolar stainless-steel stimulus electrode was placed in the Shaffer collaterals, and a glass recording electrode (blunt patch electrode) containing regular aCSF was placed in stratum pyramidale. For field EPSP recordings, electrodes were placed in stratum radiatum. A pulse (80 μs wide) was applied to elicit EPSPs. A stimulus intensity (50–200 μA) to elicit 50−60% of the maximum EPSP or population spike was used for data collection. In dentate gyrus, EPSPs were measured from lateral perforant pathway stimulation by placing the stimulus electrode and recording electrode in the outer molecular layer of the dentate gyrus, which were confirmed by paired-pulse facilitation (paired-pulse ratio 1.38 ± 0.29 at a 50-ms stimulus interval; Froc et al., 2003). To ensure that AlloP fully reached the target cells, currents, action potentials, and fEPSPs were acquired 4 min after AlloP application. Any slice with baseline drift (EPSP of last minute vs first minute) over 20% was eliminated from analysis.

### Experimental design and statistical analyses

For sIPSCs measurement, templates were first created by average >50 events, and sIPSCs were detected using the template-matching algorithm in Clampfit (pClamp suite). At least 50 events contributed to average sIPSC waveforms in each experimental condition. sIPSC decays were fit to the sum of two exponential functions, extrapolated to the peak IPSC. Decay time course is reported as a weighted time constant (τ_w_; Sun et al., 2018). For tonic GABA_A_ currents measurements, mean holding currents with 200 ms in length during baseline and each drug condition were calculated when they reached steady state. Tonic GABA_A_ currents were obtained by subtracting aCSF baseline holding currents. SD of the holding currents was calculated using Clampfit. Power analyses were used on pilot datasets to generate target N values. A paired *t* test or ANOVA as appropriate was performed using GraphPad Prism 8 or 9 to test effects within and among cells. For fEPSP recordings in CA1 stratum radiatum and dentate gyrus, the fEPSP rising slope was calculated in Clampfit. For field recordings in CA1 stratum pyramidale, the peak of the population spike was measured, or alternatively the form of EPSP was measured by the coastline burst index (CBI) as described (Widman and McMahon, 2018). Paired *t* tests were applied to compare the fEPSP rising slope or population spike during the last minute of drug application and during the last minute of baseline recording using GraphPad Prism 8 or 9. Specific analyses and exact *p* values are described in the figure legends. Statistical comparisons are presented at the level of ns not significant, *p ≤ 0.05, **p ≤ 0.01, ***p ≤ 0.001, and ****p ≤ 0.0001 in figures.

## Results

### Identification of fast-spiking PV interneurons

**Figure 2.**
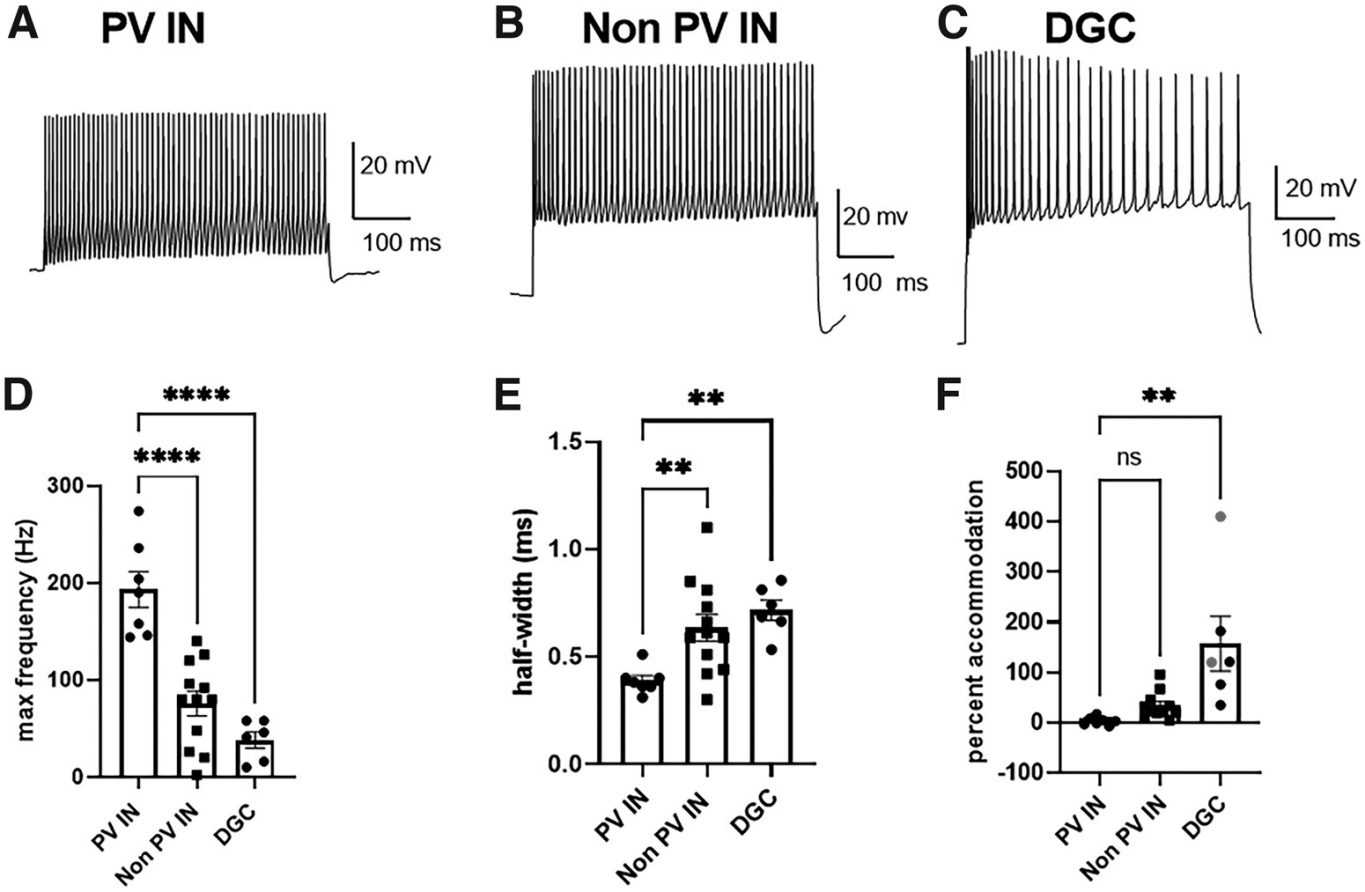
Action potential characteristics and repetitive firing patterns in fluorescent neurons readily distinguish seven PV interneurons (IN) from 12 non-PV interneurons (Non PV IN) and six DGCs. ***A–C***, Representative voltage traces induced by depolarizing current injection. ***D***, Maximum action potential frequency obtained by incremental increases in current amplitude from −50 to 400 pA. One-way ANOVA showed a cell type effect (*F*_(2,22)_ = 26.49, *p* < 0.0001). PV interneurons had a higher maximum firing frequency than non-PV interneurons and DGC (Holm–Sidak, *p* < 0.0001 for both). ***E***, Half-width of action potentials elicited by just supra-threshold current injection. One-way ANOVA showed a main effect of cell type (*F*_(2,22)_ = 7.25, *p* = 0.004). PV interneurons had a smaller half-width than non-PV interneurons and DGC (Holm–Sidak, *p* = 0.005 and *p* = 0.004, respectively). ***F***, Percent accommodation of action potentials at maximum firing frequency. One-way ANOVA showed a genotype effect (*F*_(2,21)_ = 9.431, *p* = 0.001). PV interneurons had larger accommodation than DGC (Holm–Sidak, *p* = 0.001), but not non-PV interneurons (Holm–Sidak, *p* = 0.362). The red symbols indicate two cells in which the requisite number of action potentials was not exceeded (see Materials and Methods), so the time between the final two action potentials was taken as a minimum accommodation measure.

Because PV interneurons, aside from DGCs, represent the most δ subunit-rich cell type in the hippocampus (Struble et al., 1978; Freundl and Buzsáki, 1996), we enriched for them in recording using either viral injection of pAAV5-hSyn-DIO-EGFP into hippocampi of Pv-Cre mice or the Pv-Cre line crossed to Ai14 reporter mice. A typical example of co-labeling for td-Tomato (Ai14) and PV immunostaining is shown in Figure 1 and summarized in Materials and Methods. To further confirm the phenotype of labeled cells, we characterized the firing patterns of PV interneurons. A depolarizing current was injected to elicit action potentials in labeled PV interneurons, non-PV interneurons identified either by pAAV1-mDlx-NLS-mRuby2 virus (Dimidschstein et al., 2016) or by anatomic localization (somas in the molecular layer and hilar regions of the dentate and the stratum radiatum of CA1), and DGCs, identified anatomically by somas residing in the dentate granule cell layer (Fig. 2*A–C*). As expected, PV interneurons showed fast-spiking behavior with a maximum frequency near 200 Hz (Fig. 2*D*). Also, isolated action potentials of PV interneurons were briefer than those of non-PV interneurons and DGCs, with a half-width <0.5 ms (Fig. 2*E*). Finally, PV interneurons showed little accommodation compared with the other two cell groups (Fig. 2*F*). Taken together, the results show that reporter fluorescence enriches for PV interneurons.

### AlloP promotes phasic but not tonic inhibition on hippocampal PV interneurons

**Figure 3.**
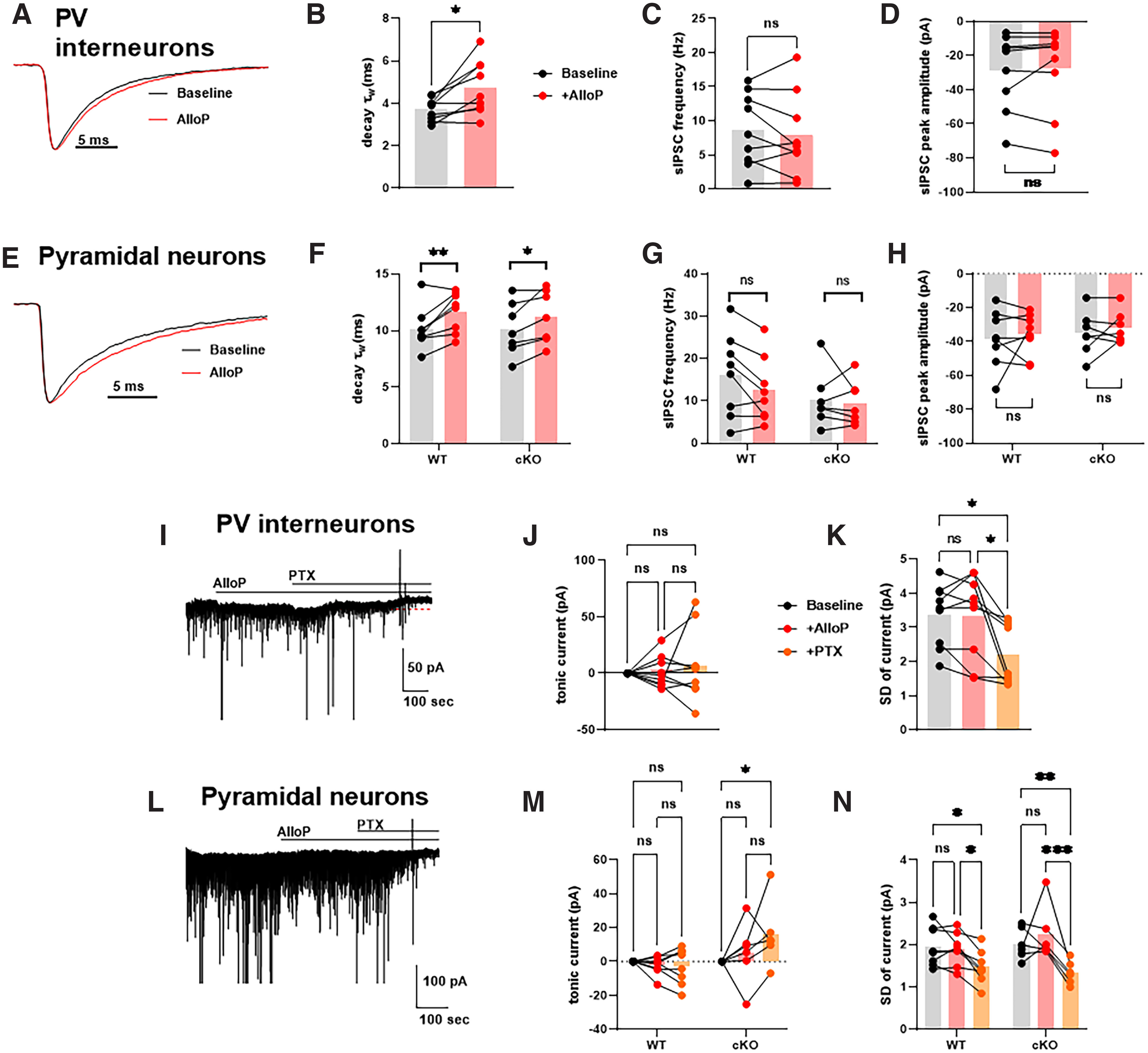
AlloP promotes phasic but not tonic inhibition in hippocampal PV interneurons and CA1 pyramidal neurons. ***A***, Representative average sIPSC waveform from a PV interneuron at baseline (black trace) and after 100 nm AlloP application (red trace). ***B–D***, sIPSC characteristics of PV interneuron (*N* = 9): (***B***) weighted decay τ (τ_w_) of sIPSC (paired *t* test, *p* = 0.015; *), (***C***) frequency (paired *t* test, *p* = 0.392; ns), and peak amplitude (paired *t* test, *p* = 0.663; ns). ***E***, Representative average sIPSC waveform from a CA1 pyramidal cell at baseline (black trace) and after 100 nm AlloP application (red trace). ***F–H***, CA1 pyramidal neuron sIPSC characteristics of WT (*N* = 8) and δ cKO (*N* = 7). ***F***, Two-way ANOVA showed a drug effect on the weighted decay τ (τ_w_) of sIPSC (*F*_(1,13)_ = 20.05, *p* = 0.0006). AlloP increased τ_w_ of sIPSCs in WT and cKO (Holm–Sidak’s test, *p* = 0.004 and 0.024, respectively). ***G***, Two-way ANOVA showed no drug effect on the frequency of sIPSC (*F*_(1,13)_ = 3.26, *p* = 0.094). AlloP had no effect on frequency of sIPSC in WT and cKO (Holm–Sidak’s test, *p* = 0.099 and 0.652, respectively). ***H***, Two-way ANOVA showed no drug effect on the amplitude of sIPSC (*F*_(1,13)_ = 0.725, *p* = 0.41). AlloP had no effect on amplitude of sIPSCs in WT and cKO (Holm–Sidak’s test, *p* = 0.76 and 0.76, respectively). ***I***, Effects of 100 nm AlloP on the holding current in a PV interneuron at −70 mV. ***J***, Summary of PV interneuron tonic current (*N* = 9). There was no discernible drug effect on the tonic current (one-way ANOVA, *F*_(1.307,10.45)_ = 0.336, *p* = 0.632). There was no difference in tonic current between baseline and AlloP, AlloP and PTX, baseline and PTX (Holm–Sidak’s test, *p* = 0.906, respectively). ***K***, Summary of the PV interneuron SD of current (*N* = 9). There was no difference in the SD between baseline and AlloP (Holm–Sidak’s test, *p* = 0.846). 100 μm PTX decreased the SD from baseline and AlloP (Holm–Sidak’s test, *p* = 0.016 and 0.028, respectively), one-way ANOVA (*F*_(1.403,11.22)_ = 10.20, *p* = 0.005). ***L***, Effects of 100 nm AlloP on the holding current in a CA1 pyramidal cell at −70 mV. ***M***, Summary of the tonic current in CA1 pyramidal cells. Two-way ANOVA showed no drug effect on the tonic current (*F*_(2,24)_ = 1.783, *p* = 0.19). AlloP had no effect on the tonic current in either WT or cKO CA1 pyramidal cells (Holm–Sidak’s test, *p* = 0.932 and 0.349, respectively). ***N***, Summary of the CA1 pyramidal neuron SD deviation of currents. Two-way ANOVA showed a drug effect on the SD (*F*_(2,24)_ =18.04, *p* < 0.0001). AlloP had no effect on the SD in either WT or cKO (Holm–Sidak’s test, *p* = 0.726 and 0.240, respectively).

To test our hypothesis of AlloP effects on hippocampal interneuron δ-containing GABA_A_ receptors, we characterized the impact of AlloP on inhibition in PV interneurons. Whole-cell patch clamp was performed on PV interneurons, and both phasic and tonic currents were measured (Fig. 3*A*,*I*). 100 nm AlloP prolonged the decay of sIPSC from 3.7 to 4.8 ms (Fig. 3*B*). Neither the peak amplitude nor the frequency of sIPSCs was altered by AlloP (Fig. 3*C*,*D*). Surprisingly, 100 nm AlloP did not induce or amplify tonic current in PV interneurons (Fig. 3*J*). Additionally, the SD of tonic current, a sensitive proxy for tonic current (Lu et al., 2020) was not increased by AlloP (Fig. 3*K*). SD of the currents was reduced by 100 μm PTX, indicating inhibition of basal GABA_A_ receptor function that was not sensitive to augmentation by AlloP (Fig. 3*K*). To summarize, AlloP potentiated the phasic inhibition on PV interneurons by increasing the decay of sIPSC, but tonic inhibition was not potentiated by AlloP.

To place these results in context, we previously found that under the same experimental conditions, AlloP potentiates tonic GABA current in DGCs (Lu et al., 2020). Combined with results mentioned above, this suggests that DGCs and PV interneurons are differentially affected by tonic inhibition and AlloP under basal conditions. Here, we extended the comparison to include CA1 pyramidal neurons. AlloP prolonged IPSC decays as for PV interneurons and DGCs (Fig. 3*E*,*F*; Lu et al., 2020). If AlloP impacts the excitability of PV interneurons through inhibition from δ-containing GABA_A_ receptors, AlloP might decrease sIPSC frequency in CA1 pyramidal cells. However, we failed to detect an effect of AlloP on CA1 sIPSC frequency (Fig. 3*G*). Furthermore, selective deletion of the δ subunit from PV interneurons (δ cKO) failed to alter the baseline frequency of sIPSCs or the effect of AlloP on sIPSC waveform in CA1 pyramidal neurons (Fig. 3*F–H*), suggesting a negligible role for PV δ-containing GABA_A_ receptors on phasic inhibition in CA1 pyramidal neurons.

Previous work has shown that tonic current in CA1 pyramidal neurons is relatively insensitive to direct neurosteroid application; THDOC at low concentration did not potentiate either phasic or tonic inhibition in CA1 pyramidal neurons (Stell et al., 2003), although effects of neurosteroids in CA1 pyramidal neurons may be sex and age dependent (Smith et al., 2009). In our experiments, AlloP did not induce detectable tonic current in CA1 pyramidal neurons (Fig. 3*L–N*). However, as in PV interneurons, PTX reduced tonic current SD with PTX application (Fig. 3*N*), suggesting a small GABA_A_R mediated conductance that proved insensitive to AlloP.

### AlloP promotes tonic inhibition of PV interneurons with exogenous GABA

**Figure 4.**
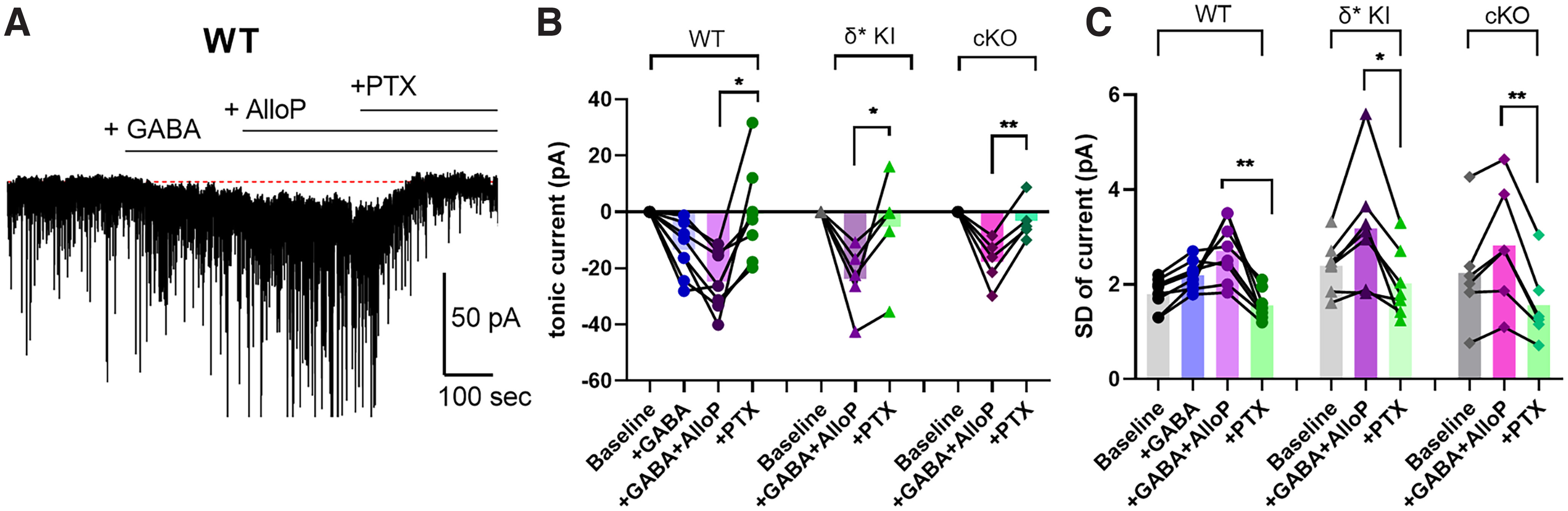
AlloP promotes tonic inhibition in the presence of 5 μm exogenous GABA. ***A***, Representative effects of 5 μm GABA, 100 nm AlloP, and 100 μm PTX on the holding current in a PV interneuron. ***B***, Summary of changes in mean current relative to pre-GABA baseline in WT, δ* KI, and δ cKO PV interneurons. In WT PV interneurons (*N* = 8), one-way ANOVA showed a drug effect (*F*_(1.942,13.6)_ = 12.79, *p* = 0.001). GABA/AlloP potentiated a tonic current of −24.8 pA, which is increased over GABA-induced current (Holm–Sidak, *p* = 0.012). There is a decrease of tonic current by 100 μm PTX (Holm–Sidak, *p* = 0.012). In δ* KI PV interneurons (*N* = 5), GABA/AlloP potentiated a tonic current of −23.8 pA. 100 μm PTX decreased GABA/AlloP potentiated tonic current (paired *t* test, *p* = 0.004). In δ cKO PV interneurons (*N* = 5), GABA/AlloP potentiated an average of −17.7 pA tonic current, which was suppressed by PTX (paired *t* test, *p* = 0.009). ***C***, Summary of the SD of current. In WT (*N* = 8), one-way ANOVA showed a drug effect (*F*_(1.330,9.311)_ = 15.98, *p* = 0.002). There is a decrease of SD by PTX (Holm–Sidak’s test, *p* = 0.016). In δ* KI PV interneurons (*N* = 7), one-way ANOVA showed a drug effect (one-way ANOVA, *F*_(1.444,8.633)_ = 11.10, *p* = 0.006). PTX decreased SD (Holm–Sidak’s test, *p* = 0.016). In δ cKO PV interneurons (*N* = 6), one-way ANOVA showed a drug effect (one-way ANOVA, *F*_(1.642,8.212)_ = 18.28, *p* = 0.001). PTX decreased SD (Holm–Sidak’s test, *p* = 0.008).

The results of Figure 3 indirectly suggest that δ-containing receptors in PV cells may not mediate effects of low neurosteroid concentrations in hippocampus. However, Figure 3 shows that there is little basal tonic current in hippocampal PV cells on which AlloP can act. Because α1/δ receptors, thought to characterize PV cells, show lower GABA sensitivity than α4/δ receptors of DGCs (Jia et al., 2005), δ-containing receptors may not contribute much to basal tonic current in PV cells. To determine whether addition of exogenous GABA might increase AlloP’s effects, we applied 5 μm GABA to enhance tonic current before application of AlloP in wild-type (WT) PV interneurons (Fig. 4*A*). GABA increased tonic current by ∼10 pA, and GABA presence unmasked AlloP potentiation of the tonic current (Fig. 4*B*,*C*, left).

To determine whether this GABA/AlloP potentiated tonic current is mediated by α1/δ receptors, we used two independent mouse lines. First, we separated tonic currents mediated by δ and non-δ receptors in PV interneurons using PTX-resistant δ* KI mice (Sun et al., 2018; Lu et al., 2020) bred to Ai14 reporter mice. In fluorescent PV cells, PTX reduced a large portion of tonic current, suggesting a contribution of non-δ receptors to the GABA/AlloP tonic current (Fig. 4*B*,*C*, middle). Furthermore, we applied GABA/AlloP in PV interneurons with δ-containing receptors conditionally knocked out (cKO). On average there was −18-pA tonic current induced in the absence of δ receptors (Fig. 4*B*,*C*, right), not different from WT PV cells. Overall, our results showed that with exogenous GABA, AlloP promotes the tonic inhibition of hippocampal PV interneurons, but the effect is not dominated by δ-containing receptors.

### AlloP decreases the firing frequency of DGCs with little impact on PVs

**Figure 5.**
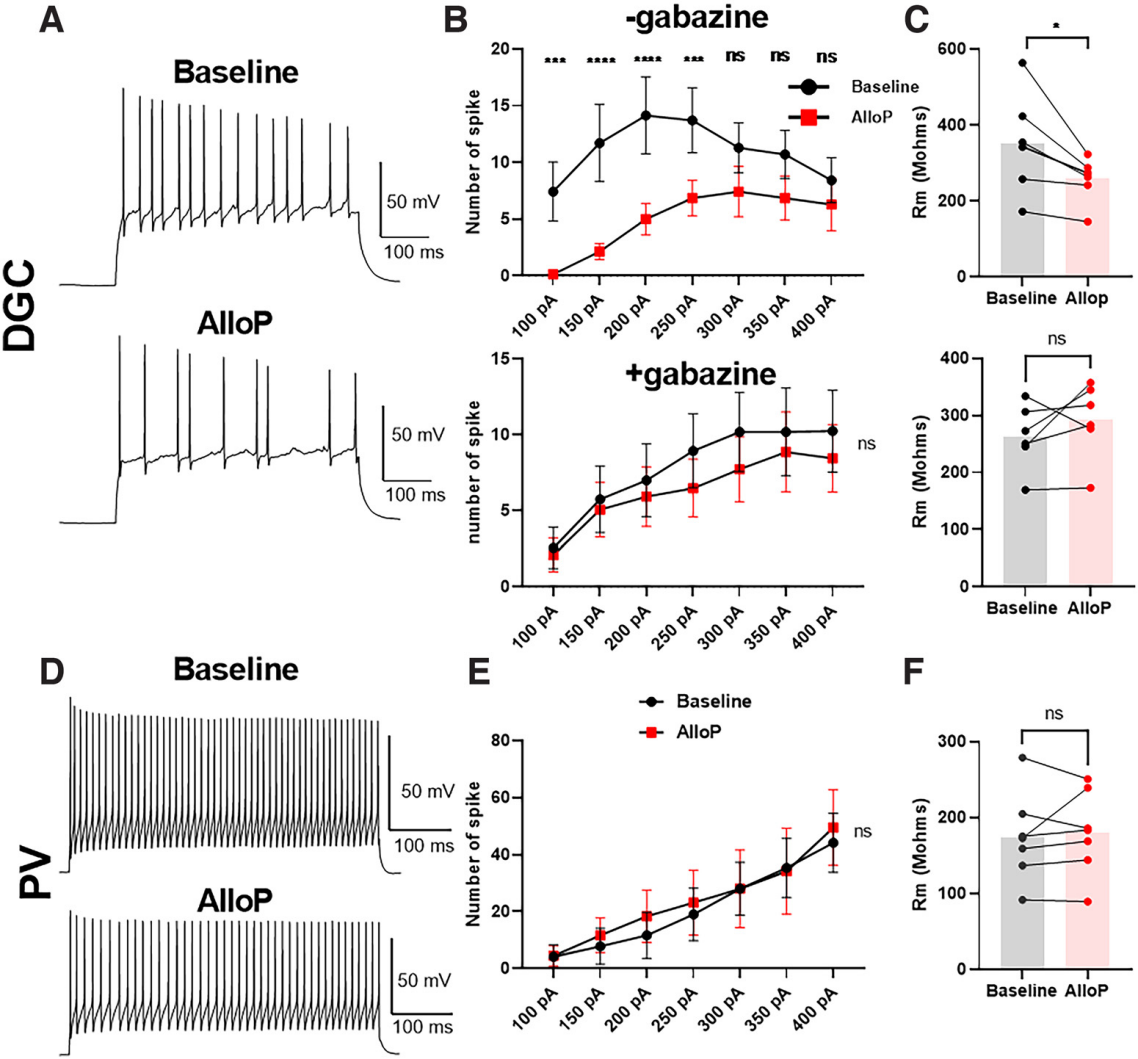
AlloP promotes inhibition of DGC firing but not PV interneuron firing. ***A***, Representative action potential patterns of DGC baseline with 200 pA current injection and following application of 100 nm AlloP. ***B***, Average number of action potentials in DGCs elicited at the indicated current amplitudes (*N* = 8). Upper panel, Two-way ANOVA on number of action potentials showed a drug effect (*F*_(1,6)_ = 10.87, *p* = 0.017). AlloP decreased the number of action potentials with 100 pA current step (Holm–Sidak, *p* < 0.001), 150 pA (Holm–Sidak’s test, *p* < 0.0001), 200 pA (Holm–Sidak’s test, *p* < 0.0001), and 250 pA (Holm–Sidak’s test, *p* = 0.0004). Lower panel, 10 μm gabazine prevented the effect of AlloP on excitability [two-way ANOVA (*F*_(6,30)_ = 1.716, *p* = 0.152)]. ***C***, Upper panel, Effect of AlloP on the DGC membrane resistance. A hyperpolarization was induced by injecting −50 pA current for 500 ms. Membrane resistance was calculated by dividing the final change in voltage by the current (*N* = 7, paired *t* test, *p* = 0.018). Lower panel, In the presence of gabazine (10 μm), there was no consistent change in the input resistance of DGCs (*N* = 6, paired *t* test, *p* = 0.277). ***D***, Representative firing of a PV interneuron at baseline and after addition of 100 nm AlloP. ***E***, Number of action potentials in PV interneurons elicited current injection (*N* = 6). Two-way ANOVA on number of action potentials showed no drug effect (*F*_(1,5)_ = 0.227, *p* = 0.654). ***F***, No effects of AlloP on the PV interneuron membrane resistance (*N* = 7, paired *t* test, *p* = 0.618). In ***D–F***, four PV interneurons were from CA1 and three were from dentate gyrus.

The results through Figure 4 suggest that AlloP may have little impact on excitability of hippocampal PV interneurons through tonic current. To test directly whether AlloP alters excitability, we administered 100 nm AlloP and measured the change of action potential firing frequency using step current injections in whole-cell, current-clamp recordings of PV interneurons. We used DGCs as comparators (positive controls) with δ-mediated tonic current that is sensitive to AlloP modulation (Fig. 5*A*,*D*). Because no synaptic stimulation was used, we expect that this protocol assays mainly the impact of tonic inhibition. We found a decrease of action potential number by AlloP in the DGCs until the maximum firing frequency was reached (Fig. 5*B*, top). Furthermore, AlloP decreased the membrane resistance of DGCs (Fig. 5*C*, top). When we applied gabazine to block GABA_A_Rs, AlloP did not alter the number of action potentials or the membrane resistance of PV interneurons (Fig. 5*B*,*C*, bottom). Our results indicate a direct inhibition of DGCs by AlloP through tonic inhibition. On the other hand, AlloP did not decrease the action potential number in PV interneurons (Fig. 5*E*). In addition, AlloP did not detectably alter the membrane resistance of PV interneurons (Fig. 5*F*). Taken together, 100 nm AlloP decreased the firing frequency of DGCs but had no impact on excitability of PV interneurons through tonic inhibition.

### AlloP potentiation of phasic inhibition does not affect the firing frequency of DGCs or PV interneurons

**Figure 6.**
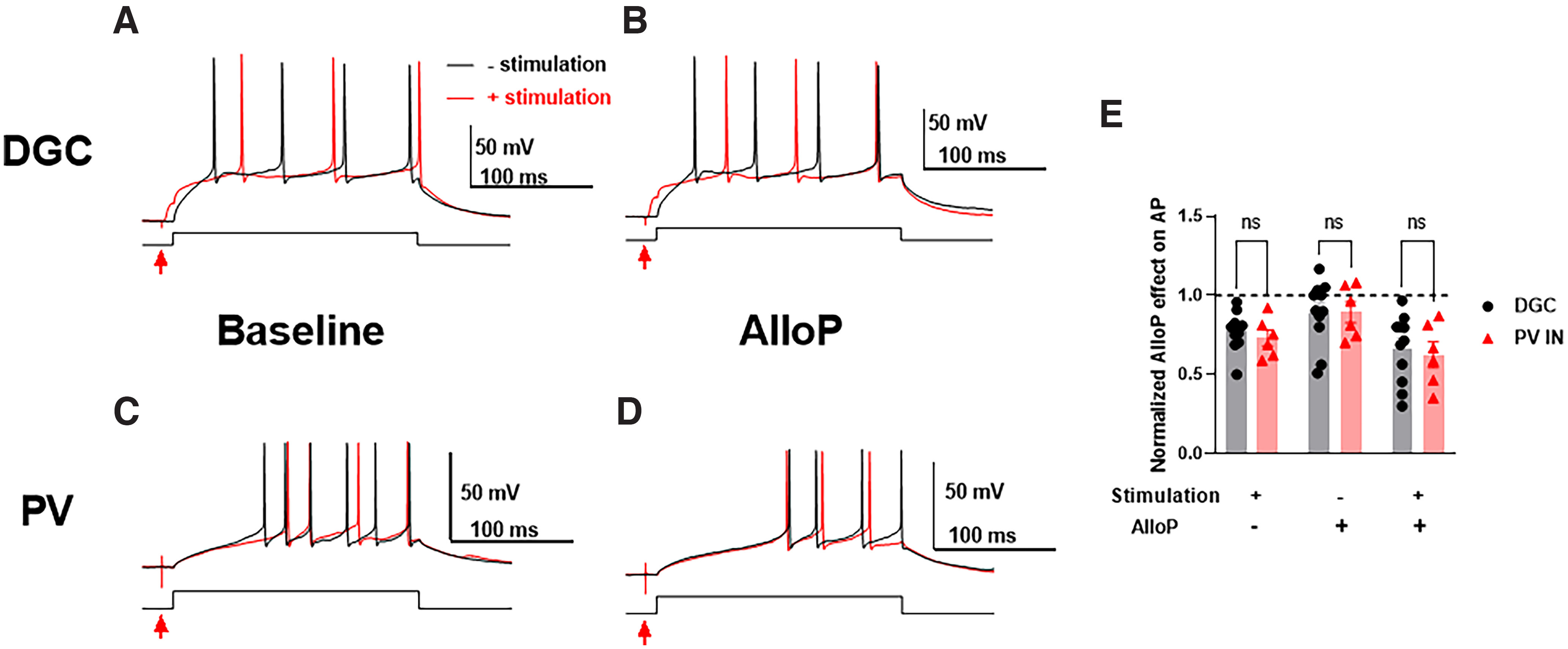
AlloP potentiation of phasic inhibition does not affect the firing frequency of DGCs or PV interneurons. ***A***, Representative action potential patterns for DGC with (red traces) or without (black traces) electrical synaptic stimulation (red arrows) during baseline or (***B***) 100 nm AlloP. ***C***, Representative action potential patterns of PV interneurons with or without electrical stimulation during baseline or (***D***) 100 nm AlloP. The first action potential latency showed no genotype difference between DGCs and PV interneurons [two-way ANOVA (*F*_(1,11)_ = 1.097, *p* = 0.3175)]. ***E***, Normalized AlloP effect on action potentials of DGCs (*N* = 11) or PV interneurons (*N* = 6). Two-way ANOVA showed no genotype difference (*F*_(1,15)_ = 0.1632, *p* = 0.6919). There is no difference for normalized action potentials between DGCs and PV INs with electrical stimulation (+), AlloP (+), or both conditions (Holm–Sidak’s, *p* = 0.955, *p* > 0.1, or *p* = 0.961, respectively).

Although AlloP has little impact on the firing frequency of PV interneurons through tonic inhibition, could the AlloP potentiation of phasic inhibition (Fig. 3) drive disinhibition of principal neurons? To test this, we injected 200-ms pulses of positive current titrated in amplitude to elicit approximately five action potentials (Fig. 6*A–D*, black traces). Control sweeps were interleaved with sweeps in which we elicited phasic inhibition with extracellular stimulation (50–100 μA) 10 ms before current injection (Fig. 6*A–D*, red traces). Excitation was left intact to allow feedback inhibition to participate. We titrated extracellular stimulation amplitude to reduce the number of action potentials slightly in both DGCs (Fig. 6*A*) and PV cells (Fig. 6*C*). However, in both cases, AlloP failed to increase phasic inhibition sufficiently to reliably alter firing (Fig. 6*B*,*D*,*E*). Furthermore, the first action potential latency showed no genotype difference between DGCs and PV interneurons. Overall, the results show that AlloP potentiation of phasic inhibition is unlikely to underlie disinhibition.

### Limited evidence of CA1 disinhibition by neurosteroids

**Figure 7.**
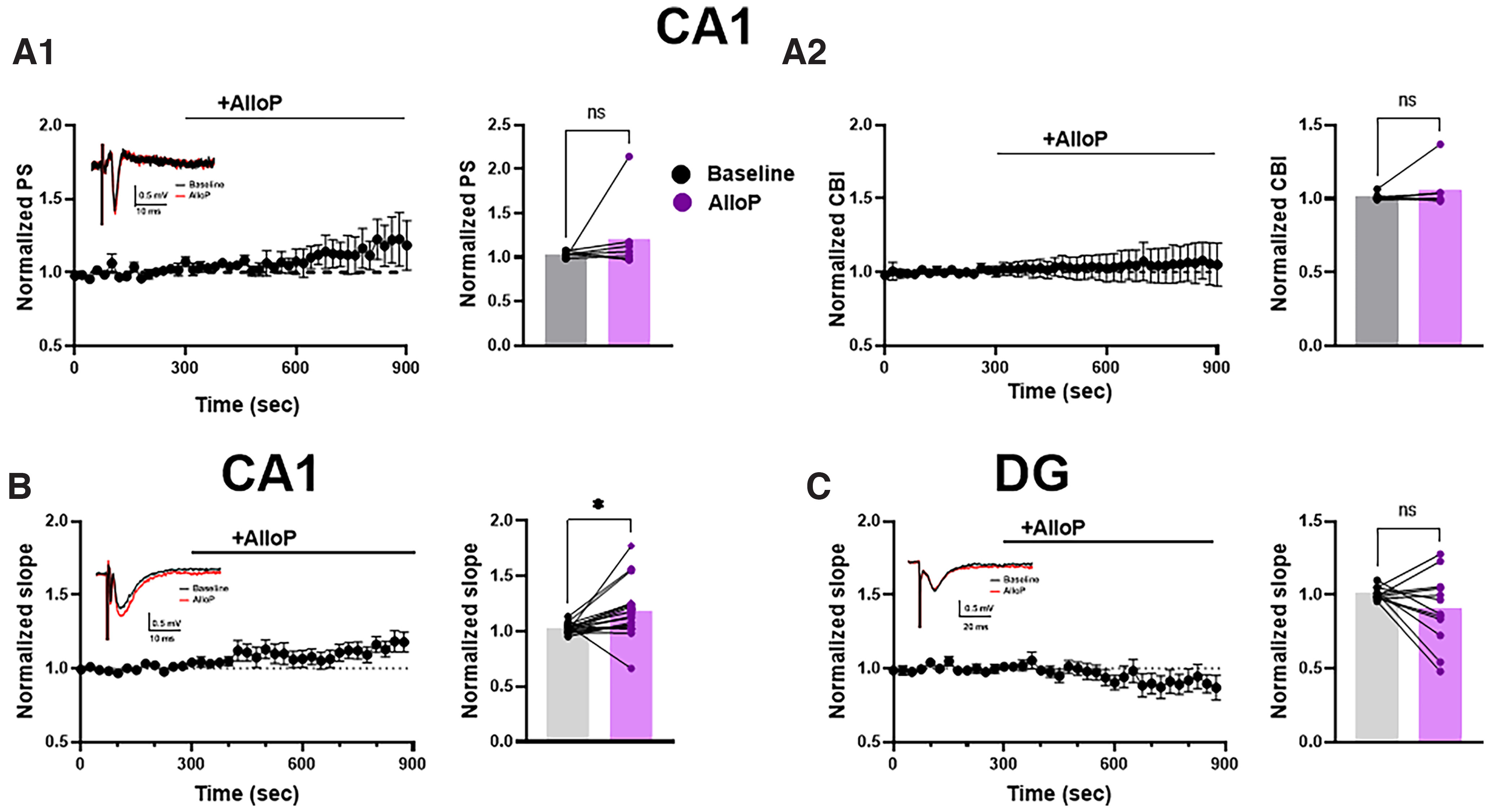
Effect of acute application AlloP on the field recordings in CA1 and DG. ***A1***, Effect of 50 nm AlloP on population spike (PS) in CA1 stratum pyramidale. PS amplitude was not altered by AlloP (paired *t* test, *p* = 0.20, *N* = 7). One additional recording was identified by the Prism outlier detector and excluded from analysis. ***A2***, Summary of effect on fields response coastal burst index (CBI) in baseline during application of AlloP in CA1 stratum pyramidale (paired *t* test, *p* = 0.24, *N* = 8). ***B***, Effect of 50 nm AlloP on rising slope of fEPSP in CA1 stratum radiatum (paired *t* test, *p* = 0.016, *N* = 17). No difference in the presynaptic fiber volley (paired *t* test, *p* = 0.234). ***C***, Effects of AlloP on rising slope of fEPSP in dentate gyrus (paired *t* test, *p* = 0.161, *N* = 12). Sample field recordings are included as insets.

Several other candidate novel antidepressants, including ketamine and scopolamine, increase pyramidal cell disinhibition in hippocampus, likely by decreasing the activity of interneurons (Wohleb et al., 2016; Widman and McMahon, 2018). We reasoned that a disproportionate effect of AlloP on GABAergic inhibition of interneurons versus principal cells could have a functionally similar effect as these other novel antidepressants, although our results suggest that tonic inhibition in PVs cells is unlikely to be an important substrate for such disinhibition. To re-investigate population excitability to synaptic stimulation, we measured the synaptic field response in the CA1 stratum pyramidale and stratum radiatum evoked via Schaffer collateral stimulation. We failed to find a reliable disinhibitory effect of 50 nm AlloP on CA1 population spikes (Fig. 7*A1*). Similarly, coastline burst index, a measure previously used to monitor ketamine’s effect on excitability (Widman and McMahon, 2018), failed to show an increase in excitability of CA1 pyramidal neurons with AlloP application (Fig. 7*A2*). On the other hand, measures of fEPSPs from dendritic field recordings revealed a small but variable increase of EPSPs by 50 nm AlloP (Fig. 7*B*). Similar results were obtained with 100 nm AlloP (1.302 ± 0.097% increase in slope, *N* = 5, *p* = 0.035).

Previous studies indicated direct inhibition from AlloP on DGCs (Stell et al., 2003; Lu et al., 2020). Consistent with an overall inhibitory effect of AlloP in dentate gyrus, we found that fEPSPs measured in the dentate molecular layer and evoked by stimulation in the lateral perforant path, failed to show evidence of enhancement (Fig. 7*C*). Taken together, the results show slightly enhanced excitatory synaptic transmission with AlloP application, an effect that does not translate into increased excitability. Overall, this observation is consistent with other results herein showing little selectivity of AlloP for δ subunit-based inhibition in interneurons.

## Discussion

In this study, we investigated the effect of the neurosteroid AlloP on tonic and phasic GABAergic inhibition in mouse hippocampal PV interneurons. Our aim was to address whether AlloP at therapeutically relevant concentrations promotes activity of principal neurons through inhibition of PV interneurons. To do that, we used genetically tagged PV interneurons in mouse hippocampus and showed that AlloP promoted phasic inhibition by prolonging the decay of spontaneous IPSCs. However, AlloP promoted tonic inhibition only when co-applied with exogenous GABA, and this potentiation of tonic inhibition was not primarily through δ-containing receptors. Furthermore, a modest AlloP concentration decreased the firing frequency of DGCs but not PV interneurons. Although AlloP modestly enhanced fEPSPs from CA1 pyramidal neurons, this effect did not translate into enhanced CA1 excitability. Thus, neither effects at the cellular nor population level indicate that hippocampal PV interneurons are a likely substrate for therapeutic AlloP effects.

### AlloP promotes phasic but not tonic inhibition on PV interneurons

The role of δ-containing GABA_A_ receptors in the effects of AlloP remains of interest. A previous study showed that AlloP potentiated phasic inhibition in DGCs mainly through γ2-containing receptors (Lu et al., 2020), which greatly outnumber δ-containing receptors in DGCs (Sun et al., 2018). PV interneurons also express γ2-containing receptors, and these receptors should be the primary substrate of the AlloP effect on phasic inhibition in these interneurons. Surprisingly, at concentrations that prolonged phasic inhibition, AlloP did not promote tonic inhibition in PV interneurons, which we initially presumed was driven by α1/δ-containing GABA_A_Rs. Previously, it was shown that α1/δ-containing GABA_A_Rs have low GABA potency and efficacy (Jia et al., 2005; Jensen et al., 2013), which could explain the weak AlloP effects in PV interneurons. When AlloP was co-applied with GABA, the promotion of tonic inhibition in dentate gyrus interneurons was much lower than that in DGCs (Carver and Reddy, 2016). Indeed, AlloP potentiated tonic current in PV interneurons only when exogenous GABA was applied (Fig. 4*A*,*B*). However, when we blocked non-δ containing receptors using PTX in δ* KI PV interneurons, we eliminated a large amount of tonic current potentiated by GABA/AlloP (Fig. 4*B*,*C*, middle), suggesting non-δ receptor contributions. Similarly, in cKO PV interneurons, GABA/AlloP potentiated smaller but large portion of tonic current compared with those in WT (Fig. 4*B*,*C*, right). Both our KI and cKO data indicate a contribution of non-δ receptors to GABA/AlloP potentiated tonic current. However, another study showed that in basolateral amygdala, AlloP promoted tonic inhibition in PV interneurons, but not in principal cells, through δ-containing receptors (Antonoudiou et al., 2022). This may indicate differences in subunit composition, post-translational modification of receptors, or GABA levels for PV interneurons in different brain areas important for neuropsychiatric symptoms. One mystery in our observations is the nature of baseline PTX-sensitive tonic current that is insensitive to AlloP (Fig. 3*J*,*K*). Because AlloP is a broad-spectrum modulator of GABA_A_ subclasses, it is surprising that this current was unaffected by AlloP.

AlloP potentiates both phasic and tonic inhibition in DGCs (Lu et al., 2020). The tonic current promoted by AlloP inhibits DGCs, increases membrane conductance, and thus reduces neuronal excitability (Fig. 5*B*,*C*). On the contrary, AlloP failed to alter excitability of PV interneurons (Fig. 5*E*,*F*), consistent with the weak effect on tonic current. It may be noteworthy that the experiment in Figure 6 failed to exhibit a tonic effect of AlloP even on DGCs, in contrast to Figure 5. We attribute this discrepancy to the briefer stimulus used to elicit action potentials in Figure 6; AlloP’s tonic effects on spiking were most evident late in action potential trains in Figure 5. Regardless, neither experimental protocol revealed a detectable effect of AlloP on tonic inhibition in PV neurons.

### AlloP promotes dendritic fEPSPs in CA1

The rapid antidepressant ketamine at subanesthetic concentrations enhances population responses in hippocampal CA1 (Widman and McMahon, 2018). The promotion of dendritic fEPSPs by AlloP (Fig. 7*B*) indicates that AlloP could decrease tonic or feed-forward phasic inhibition from interneurons to disinhibit principal cells (Hasselmo and Schnell, 1994; Chapman et al., 1998). However, this effect did not translate into a reliable effect of AlloP on excitability measured in CA1 population spikes (Fig. 7*A1*,*A2*). Overall, the results are consistent with little to no selectivity of AlloP on inhibitory interneuron populations. The difference in fEPSP sensitivity to AlloP by hippocampal subregion might be explained by the different GABA_A_R subunit composition in DGCs versus CA1 pyramidal cells and by the strong overall effect of AlloP on DGCs (Lu et al., 2020) compared with interneurons (present work).

The enhanced fEPSPs in CA1 could suggest an AlloP effect on a different interneuron population, such as cholecystokinin or somatostatin interneurons, although we failed to detect a change in sIPSC frequency on CA1 pyramidal cells (Fig. 3*F*). Alternatively, AlloP could directly target glutamate transmission to promote modest fEPSP enhancement. Although fiber volleys were not altered, presynaptic calcium influx or AMPA receptor sensitivity could be affected.

In summary, we examined the effect of AlloP on both phasic and tonic GABAergic inhibition in PV interneurons. Our results showed that AlloP potentiated phasic inhibition through GABA_A_Rs. However, AlloP potentiated tonic inhibition only with exogenous GABA added, and this AlloP potentiation of tonic inhibition is not primarily through δ-containing receptors. Furthermore, we showed AlloP had little effect on the excitability of PV interneurons. Although AlloP modestly enhanced fEPSPs from CA1 pyramidal neurons, this effect did not translate into enhanced CA1 excitability. Taken together, our work suggests that PV interneurons seem an unlikely substrate for AlloP-induced network effects through GABA_A_ receptors, particularly those containing a δ subunit. Other mechanisms remain to be explored to account for common antidepressant benefits of AlloP.

## Synthesis

Reviewing Editor: Jean Christophe Poncer, INSERM Decisions are customarily a result of the Reviewing Editor and the peer reviewers coming together and discussing their recommendations until a consensus is reached. When revisions are invited, a fact-based synthesis statement explaining their decision and outlining what is needed to prepare a revision will be listed below. The following reviewer(s) agreed to reveal their identity: Paul Davies. Note: If this manuscript was transferred from JNeurosci and a decision was made to accept the manuscript without peer review, a brief statement to this effect will instead be what is listed below.

Both reviewers recognize the importance and timeliness of your work, aimed at understanding the mode of action of allopregnanolone on the hippocampal circuit. They also recognize that information regarding its specific effects on PV interneurons was lacking. However, they also have some issues with the data and its interpretation that will need to be resolved before considering publication in eNeuro.

Collectively, we agreed that the following issues should be addressed as a priority:

1. The manuscript aims to assess the effects of allopregnanolone on PV interneurons in the dentate gyrus and CA1 area and compare them to those in principal neurons. However, the data are not always presented in a way that allows such a comparison. In particular, the effect on CA1 pyramidal cells is only shown in Figure 8 when it should be compared to those on PV cells shown in Figure 3. Data on DG granule cells are not available. Therefore, the statement that PV interneurons “possess low sensitivity to AlloP” seems partially unfounded. A more systematic comparison and presentation of data from the different cell types side by side in a single figure is needed.

2. We are also concerned about the variability in experimental conditions, particularly the sex and age of the animals. Given the influence of age and sex on the effects of neurosteroids, we believe this issue needs to be addressed. We suggest that statistical analysis be performed separately on data from males versus females on the one hand, and adolescent versus adult mice on the other. However, the number of observations of these different conditions may not be sufficient to conclude on the impact (or lack thereof) of these parameters on the observed effects and further recordings may be necessary.

3. In experiments testing the impact of AlloP on neuronal excitability, we believe that the link to changes in synaptic inhibition is missing. Therefore, the claim that “AlloP promotes inhibition of DG granule cell firing” is not supported by your data. We are confident that AlloP affects DG granule cell firing, but not that this is due to a change in inhibition. This needs to be clarified by additional recordings that could include repeating these experiments while pharmacologically blocking GABAAR-mediated inhibition.

---

Reviewer #1

The authors address a very interesting topic, the mechanisms of action of the fast antidepressant compound AlloP. They use different electrophysiological techniques to study excitability and GABAergic signalling in PV INs, granule cells and CA1 pyramidal neurons. They test the hypothesis that AlloP effects on PV INs alter hippocampal circuits through disinhibition, based on specific GABAR submit composition in this inhibitory cell type. On view of the relatively modest sensitivity of PV INs to AlloP, they conclude that they are unlikely contributors to the mood altering properties of neurosteriods.

In my opinion, the conclusions strongly rely on a set of negative results whose interpretation is hindered by the lack of adequate positive controls of AlloP synaptic and network actions, direct comparison of AlloP effects on GABA signalling in different types of hippocampal neurons and direct evidences of the effect of PV INs in the excitability of the dentate gyrus and CA1 regions. I have some suggestions that may help to strengthen the conclusions raised by the authors.

Major.

1. The authors demonstrate that AlloP increases sIPSCs decay time in PV INs (Fig. 1) and CA1 pyramidal neurons (Fig 8). A direct comparison of that effect in DG GCs, PV INs and CA1 pyramidal neurons would help to support the claim that PV INs “possess low sensitivity to AlloP”. Moreover, in light of the particular composition of GABA R present in PV INs (Delta-alpha1), it seems interesting to determine the effect of AlloP in mice with deletion of the delta subunit (akin to experiments in CA1 pyramidal neurons, Fig. 8).

2. From the data included in Fig. 4, authors conclude that AlloP increases tonic inhibition in the presence of 5uM GABA. However, this effect is not reflected in the statistical analysis due to the lack of direct comparison between GABA and GABA+AlloP groups. The identification of the composition of the GABA R involved (not containing the delta subunit) could seed light on the mechanism of action of AlloP in PV INs and excitatory neurons.

3. There is not a clear link between AlloP decreased excitability and GABA signalling in GCs (Fig. 5, 6), that may be tested by repeating the experiments in the presence of GABAR blockers. This limits the conclusion of the experiments performed in PV INs.

4. To obtain a clear interpretation of field experiments in Fig. 7, the effect of targeted manipulation of PV INs or principal neurons in CA1 and DG should be determined.

5. The authors used male and female mice in a range of age (P30-P60) that includes adolescence. Despite the well known effects of age and sex in neurosteriod effects (acknowledged by authors, line 268), this aspect is not taken into account in the data description and analysis.

Minor.

1. The statements in the introductions lines 54 and 55 would need references to support them.

2. Line 316 makes references to panels in G and H of Fig. 3 while this figure only includes panels A-F.

--

Reviewer #2

The existence of GABA(A)Rs consisting of a1bd subunits in interneurons have largely been ignored. When the topic of neurosteroid modulation of GABA(A)Rs has been discussed it is primary been focused on GABA(A)Rs in pyramidal neurons and little attention or thought has been paid to receptors in interneurons.

Activity of GABA(A)Rs in interneurons of the hippocampus have been demonstrated to influence the excitability of interneurons, low conductance increasing intraneuronal activity and higher conductance inhibiting interneuron excitability which, in turn, would have an impact on the excitability of pyramidal neurons (Pavlov et al., 2014). The current manuscript seeks to investigate the contribution of delta subunit containing GABA(A) receptors in PV interneurons and how they impact the excitability of hippocampal pyramidal neurons.

Abstract, page 1, line 16-18, Discussion, page 14, line 326-7, page 15, lines 350-352. The summary conclusions are too broad. The authors demonstrated that in the presence of GABA, AlloP did affect PV tonic current. They did not investigate that this GABA-dependent property of AlloP modulated overall PV and hippocampal excitability and hence can not rule out that PV interneurons are the substrate for AlloP mediated mood-altering effects. Brain slices successfully perfused in an experimental set up may require additional GABA to recapitulate a physiological environment. However, even with added GABA the authors demonstrated that the delta subunit was highly unlikely to contribute to AlloP modulation of tonic current in PV interneurons. Hence, please change the appropriate summaries of the study that more specifically state that it is the delta subunit containing GABA(A) receptors in PV interneurons that are unlikely to contribute to network excitability and antidepressant effects of AlloP.

Abstract, page 1, lines 14-16. “...impact on PV interneurons was not stronger”. Stronger than on DGCs? Please reword the sentence.

Introduction, page 2, line 34. High GABA affinity. Please list a reference that demonstrates high GABA affinity. The quoted references show shifts in GABA responses in electrophysiology experiments. But such shifts can be a combination of agonist affinity, potency, and efficacy and not a measure of how well GABA binds to the receptor.

Methods. How long was AlloP applied before currents, APs, and EPSPs were measured?

Results and Figures 3 & 4. In Fig 4 +GABA+AlloP is demonstrated to be significantly different to +PTX demonstrating the allosteric effect of AlloP in the presence of GABA in WT, d* KI and d cKO mice. But in Fig 3E&F only the stats of baseline vs +AlloP and baseline vs +PTX are shown. To make the analysis consistent the statistical comparison of +AlloP and +PTX should be shown. This could impact the conclusions drawn.

Results, page 11. When examining CA1 disinhibition the authors used 50 nM AlloP. The other data in the manuscript used 100nM. This makes it difficult to compare data so the CA1 disinhibition experiment should be repeated with 100nM AlloP or at least explain why a lower concentration is used.

Discussion, page 13, line 309. “..GABA/AlloP potentiated smaller but large portion of tonic current...” please make clearer what smaller but large means.

Discussion, page 13, line 316. Fix “Fig 3 G,H”

Discussion, page 13, line 320. The authors did not demonstrate that the tonic current hyperpolarizes DGCs. Tonic current can be depolarizing and still inhibitory due to the conductive nature of this type of inhibition. Either show that it hyperpolarize or remove the word.

Throughout the manuscript please be specific when it comes to phasic and tonic currents that they are from DG, CA1, or PV cells.

## Author Response

We apologize for the oversight. We have adjusted the Methods wording to indicate that male mice

were used in the main analysis.
